# Water-soluble gadolinium fullerenes Gd@C_82_-TEGs as a potential magnetic resonance imaging contrast agent

**DOI:** 10.1371/journal.pone.0346592

**Published:** 2026-04-10

**Authors:** Wanyun Liu, Ping Huo, Xiuming Zhou, Dayan Xiong

**Affiliations:** Yichun University, Yichun, Jiangxi, China; National University of Ireland, Galway, IRELAND

## Abstract

The water-soluble gadolinium fullerenes Gd@C_82_-TEGs nanoparticles as a novel magnetic resonance imaging (MRI) contrast agent were fabricated by the metal fullerenes Gd@C_82_ assembled with tetraethylene glycol (TEG). The loading Gd amount in Gd@C_82_-TEGs nanoparticles was 8.0 wt%. The average particle size of water-soluble Gd@C_82_-TEGs nanoparticles was 97 nm and the nanoparticles were uniformly spherical shape and had good dispersibility and no aggregation. The 97 nm spherical shape of Gd@C_82_-TEG nanoparticles ensured high relaxivity. The proton relaxivity (*r*_*1*_) and transverse relaxivity (*r*_*2*_) were high up to 37.1 mM^−1^·s^−1^ and 64.9 mM^−1^·s^−1^, which greater than that of commercial Magnevist under the same conditions. The Gd@C_82_-TEGs nanoparticles exhibited pronounced *T*_*1*_-weighted MRI *in vitro*. The MRI signal-enhancing efficiency of Gd@C_82_-TEGs nanoparticles was superior to that of gadolinium diethylenetriamine pentaacetate Gd-DTPA (Magnevist). The Gd@C_82_-TEGs nanoparticles had no significant effect on cell metabolism and structure, and the survival rate of cells was more than 80%, which indicated that the as-prepared Gd@C_82_-TEGs nanoparticles were no cytotoxicity.

## Introduction

Magnetic resonance imaging (MRI) is a high-resolution imaging method without injury and ionizing radiation, which plays an important role in clinical diagnosis, especially in cancer and tumor detection [1–5]. However, due to the lower sensitivity of MRI, about 40% ~ 50% of MRI diagnosis requires the use of MRI enhancers (contrast agents) at present. These contrast agents usually are paramagnetic substances, which can influence the water quality sub-relaxation time in local tissues of the body and change the signal intensity, lead to produce an angiographic effect [6]. The MRI contrast agents used in clinic are mainly gadolinium (Gd) chelating agents with small molecular weight, for example gadolinium diethylenetriamine pentaacetate benzyl methyl amide Gd-DTPA-BMA (Omniscan) and gadolinium diethylenetriamine pentaacetate Gd-DTPA (Magnevist) [7,8]. However, these contrast agents are limited to extracellular fluid contrast and have low relaxation efficiency and poor tissue targeting. The chelation of Gd^3+^ ions with ligands lead to the decrease of relaxation ability, and even under some specific conditions, the chelate will release Gd^3+^ to produce toxicity [9–13]. In addition, for some small tissues or even single cells, contrast agents with higher relaxation efficiency were needed. Hence, the exploration of novel contrast agents which meet with efficient, low toxicity and tissue specificity is important to make early diagnosis with pathological tissues.

Gadolinium fullerenes refer to a kind of embedded metal fullerenes with gadolinium atoms or clusters embedded in carbon cages. These fullerenes not only possess the paramagnetic properties of embedded gadolinium, but also maintain the properties of carbon cages, such as large specific surface area, stability and easy to be multi-functional and so on [14]. As a novel MRI molecular imaging probe, the relaxing mechanism of gadolinium fullerenes toward water molecules is different from the traditional gadolinium chelate. The embedded gadoliniumatoms or gadolinium clusters can indirectly relax water molecules through the outer carbon cage. The carbon cage has a large area of action, high efficiency and the dipole-dipole interaction between molecules, which further improves gadolinium fullerenes’ relaxation efficiency. More than that, the embedded clusters could be protected through fullerenes. Because the carbon cages of fullerenes can prevent the attacking of metabolic products and leakage. Therefore, biological safety of gadolinium fullerenes is greatly improved [15]. However, the poor water solubility of metal fullerenes often limits its application in biomedicine. Therefore, research and preparation of water-soluble metal fullerene derivatives comes to be the primary problem that needs to be solved. The hollow carbon-cage structure of fullerenes can not only achieve good inclusion of gadolinium atoms, but also the surface structure of fullerenes can be easily modified a variety of water-soluble fullerenes. There were a few methods about surface modification, such as hydroxylated, carboxylated, PEGylated and so on [16]. A series of water-soluble lanthanide fullerenes as new magnetic resonance contrast agents were reported by Kato [17]. Compared with the water-insoluble Gd-DTPA, the relaxation ability of Gd@C_82_(OH)_n_ (n=30~40) has even reached 20 times of Gd-DTPA. Because Gd-based metallofullerenes had unique advantages, more and more new water-soluble gadolinium fullerene derivatives had been found, such as Gd@C_82_(OH)xOy [18,19], Gd@C_60_[C(COOH) _2_]_10_ [20,21], Gd_3_N@C_80_[DiPEG(OH)_x_]_13_ [22] and so on. These water-soluble gadolinium fullerene derivatives exhibited great effect in biological imaging. Different gadolinium fullerenes derivatives exhibited varying relaxation rates. Compared to Gd@C_60,_ Gd@C_82_ was widely believed to possess superior relaxation properties due to its higher electron spin density and unique electronic structure. The hydroxyl functionalized gadofullerenes show significantly higher relaxivities than those of carboxylic derivatives [20,21].The hydroxylation modification of hemiketal structure Gd@C_82_(OH)xOy leaded to instability in its chemical structure, and the difference in the number of hydroxyl groups in the structure may result in diversity in relaxation rates [18].

To enhance the relaxation rates of gadolinium fullerenes, a novel MRI contrast agent water-soluble gadolinium fullerene nanoparticles Gd@C_82_-TEG with clear structure successfully were synthesized through a simple self assembly method. The chemical composition, particle size and morphology of these nanoparticles were analyzed and characterized by X-ray photoluminescence spectroscopy, laser nano-particle size instrument and transmission electron microscope. The Gd@C_82_ core coupled with the neutral and hydrophilic surface provided by TEG, exhibited high relaxivity, pronounced *T*_*1*_-weighted MRI *in vitro* and no cytotoxicity.

## Materials and methods

### Materials

Gd@C_82_ fullerene was purchased from Xiamen Funano New Material Technology Co. Ltd. DMEM (dulbecco#39;s modified eagle medium) and Newborn Calf serum were purchased from Gibco. Human breast cancer cells were newly purchased from the Shanghai cell center.

### Preparation of Gd@C_82_-TEGs nanoparticles

**Fig 1:**
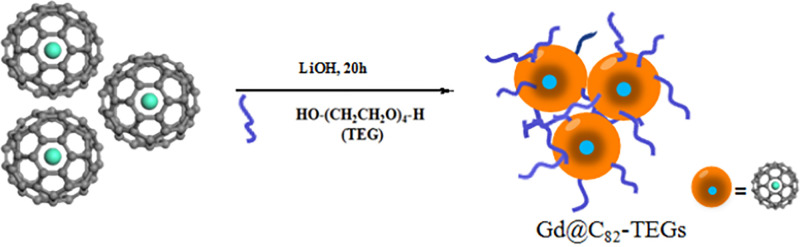
The preparation of Gd@C_82_-TEGs nanoparticles.

The Gd@C_82_-TEGs nanoparticles powders were obtained by the reaction of Gd@C_82_ fullerene solution [23–25] and tetraethylene glycol (TEG) under lithium hydroxide as catalyzer (Fig 1). The Gd@C_82_ fullerene solution (a solution of 1 mg/mL in 20 mL toluene), tetraethylene glycol (TEG, 20 mL) and lithium hydroxide(LiOH) (20 mg) were added into the reaction bottle in order. After reaction 20 hours, through adding excess ethyl acetate (EA), the obtained fullerene nanoparticles were precipitated. The obtained precipitate was dissolved in 20 mL of ultrapure water (18.2 MΩ·cm, 25^o^C), and the solution was loaded into a dialysis bag (molecular weight cutoff: 3.5 kDa). Dialysis was performed with ultrapure water for 48 h, resulting in a clear and transparent solution. The aqueous solution appeared darker. The post-dialysis solution was filtered through a 0.45 µm membrane filter. The filtrate was pre-frozen at −20 °C in a refrigerator, followed by lyophilization for 24 h to yield a brown powder. The powder was further dried for 48 h in a vacuum oven at 25 °C, then stored in a desiccator at ambient temperature.The weight of obtained Gd@C_82_-TEGs nanoparticles powders was 30 mg.

### Characterization of Gd@C_82_-TEGs nanoparticles

At room temperature, 5 mg of Gd@C_82_-TEGs nanoparticles was dissolved in redistilled water, ultrasonically dispersed homogeneously, and diluted. The particle size and distribution of the nanoparticles were measured using a laser nanoparticle size analyzer Nano-ZS90 at a wavelength of 633 nm.

The microscopic morphology of Gd@C_82_-TEGs nanoparticles were measured through transmission electron microscopy (TEM) (JEOL-2100F) at 150 kV. Samples for TEM were prepared by directly dropping Gd@C_82_-TEGs nanoparticles aqueous solution onto carbon-coated copper grids, dyeing with 2% phosphotungstic acid solution and drying at room temperature.

The chemical composition of Gd@C_82_-TEGs nanoparticles were analyzed using X-ray photoluminescence spectroscopy (XPS). The X-ray photoelectron spectroscopy (XPS) was performed on Thermo K-Alpha, operating at 12 kV and 6 mA with an alumina target (Al kα, *hv* = 1486.68eV).The amount of Gd loaded on Gd@C_82_-TEGs nanoparticles was determined by inductively coupled plasma-atomic emission spectrometry (ICP-AES, OPTIMA 5300DV).

The longitudinal (*T*_1_) and transverse (*T*_2_) relaxation times of the Gd@C_82_-TEGs nanoparticles were determined by a 1.5 T clinical MR instrument (Sonata Siemens) at 37 °C by the *T*_1_-weighted spin–echo method. The *in vitro* MRI phantomimages were acquired using a MicroMR-25 mini MR system (NiumagCorporation, Shanghai, China). The measurement parameters were as follows: *T*_1_-weighted sequence, spin echo, TR/TE = 600/18.125 ms, matrix acquisition = 128 × 128, NS = 2, FOV = 100 mm × 100 mm, Slice Thickness/Gap = 3 mm/0.5 mm, 0.50 T, 32.0 °C. The values of *T*_1_ and *T*_2_ were determined by inversion-recovery and Carr-Pucell-Meiboom-Gill method respectively. Through the fitting of the 1/*T*_1_ and Gd^3+^concentration, the *r*_1_ value was obtained. Consistent with the above method, the *r*_2_ value was obtained by fitting between 1/*T*_2_ and Gd^3+^concentration.

### *In vitro* cytotoxicity of the Gd@C_82_-TEGs nanoparticles

A standard MTT (3-(4,5-dimethylthiazolyl-2)-2,5-diphenyl tetrazolium bromide) assay was conducted using a human breast cancer cell line (MCF-7) to estimate the *in vitro* cytotoxicity of Gd@C_82_-TEGs nanoparticles. The preparation of MCF-7 cells and disinfection of Gd@C_82_-TEGs nanoparticles were referred to the previous work [26].

Inoculate prpared MCF-7 cells into a 96 well plate with a cell density of 6 × 10^5^ cells per well, and incubate in a moist atmosphere with 5% CO_2_ in 37 °C culture medium for 24 hours. Then take out the culture medium and mix the cells with those containing 100 μL Gd@C_82_-TEGs nanoparticles with different concentrations (0, 25, 50, and 100 mg/L). The mixture were continued to incubate at the above same condition for 48 hours.

The cell viabilities of Gd@C_82_-TEGs nanoparticles against MCF-7 cells were assessed using MTT. The concrete method was referred to reference [8].

## Results and discussion

### Characterization of Gd@C_82_-TEGs nanoparticles

The water-soluble Gd@C_82_-TEGs nanoparticles as a novel MRI contrast agent were synthesized via the metal fullerenes Gd@C_82_ assembled with tetraethylene glycol (TEG) for 20 h (Fig 1). Lithium hydroxide acts as a base catalyst that promotes cage-opening of C_82_ via nucleophilic attack, facilitating the incorporation of oxygen into the π-conjugated framework to form oxygen-functionalized derivatives.

**Fig 2:**
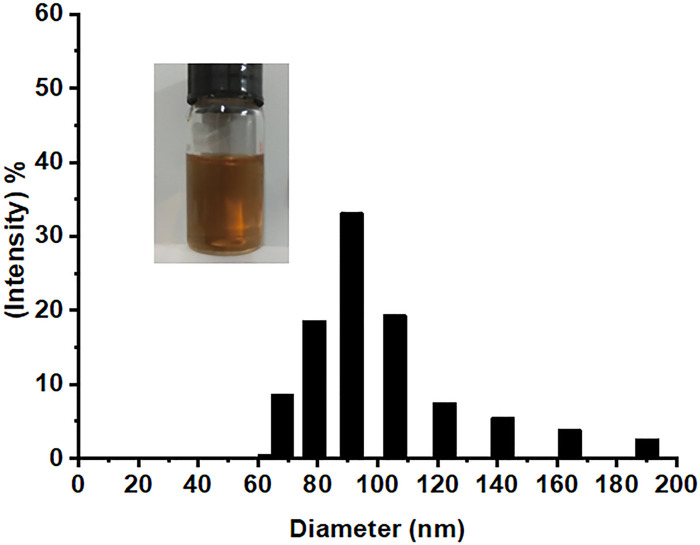
The particle size of Gd@C_82_-TEGs nanoparticles.

The particle size and distribution of Gd@C_82_-TEGs nanoparticles were determined by dynamic light scattering (DLS). Fig 2 showed the particle size distribution of Gd@C_82_-TEGs nanoparticles. The average particle size of Gd@C_82_-TEGs nanoparticles was 97 nm. The size of nanoparticles directly affects their longitudinal relaxivity and *in vivo* clearance pathways. It is well known that nanoparticles <5 nm are rapidly cleared by renal filtration, limiting imaging duration, whereas those >100 nm are sequestered by liver and spleen macrophages, impairing target accumulation. The average particle size of Gd@C_82_-TEGs nanoparticles could effectively evade rapid clearance by the reticuloendothelial system, prolong circulation time, enhance accumulation in tumor tissues via the enhanced permeability and retention effect, and thereby achieve high relaxivity and prolonged circulation.

**Fig 3:**
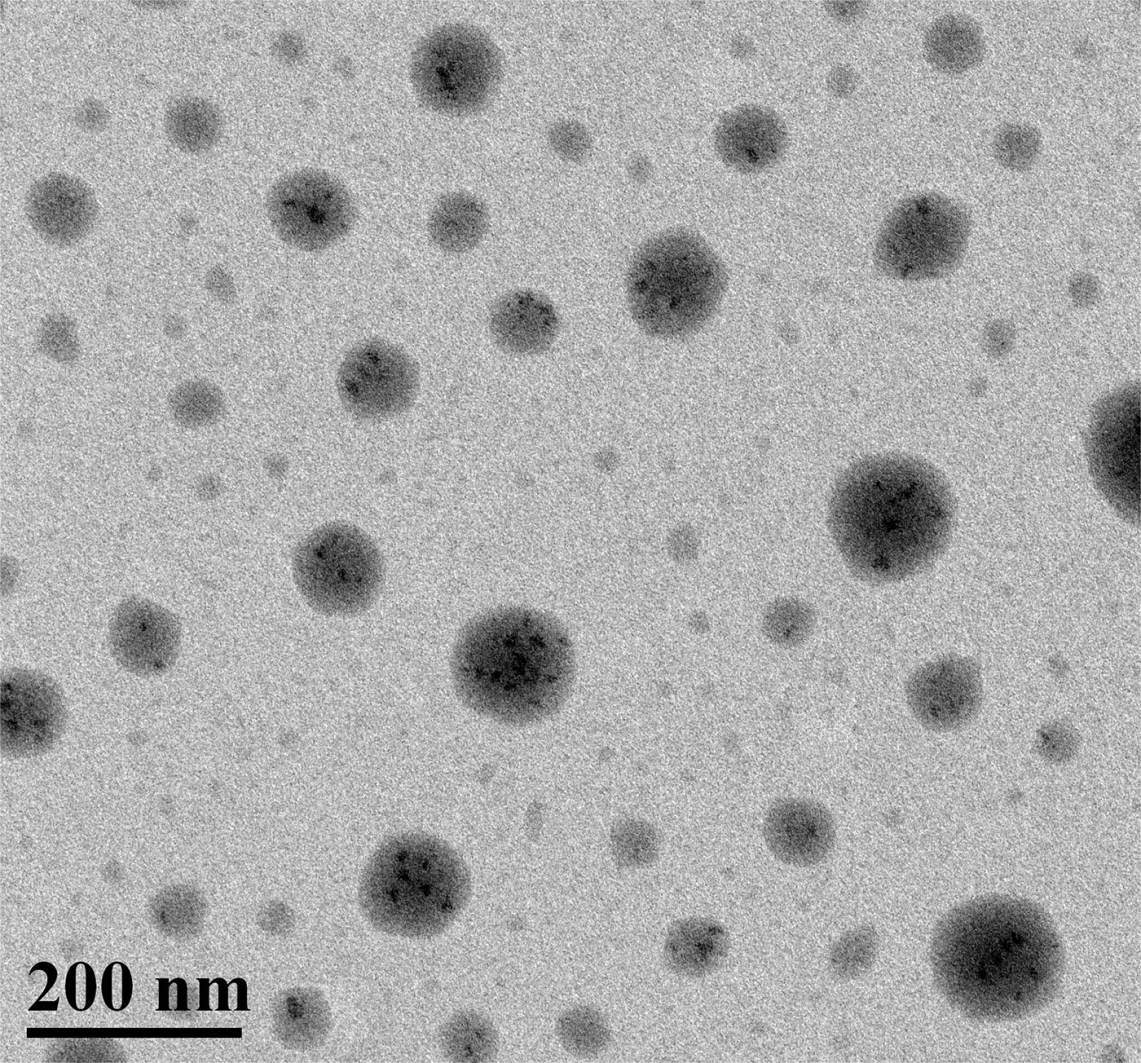
The TEM image of the Gd@C_82_-TEGs nanoparticles.

The morphology of the nanoparticles was observed by TEM in Fig 3. The nanoparticles with spherical distribution were observed and had good dispersibility and no aggregation. The spherical configuration ensured complete encapsulation of Gd³ within the C_82_ carbon cage, preventing the leakage of free gadolinium and reducing the risk of nephrogenic systemic fibrosis. [27] From the enlarged electron micrograph, it can be seen that small black metal clusters are gathered inside the carbon cage structure of fullerene. The average size of nanoparticles was about 100 nm, which was consistent with the analysis of DLS.

**Fig 4:**
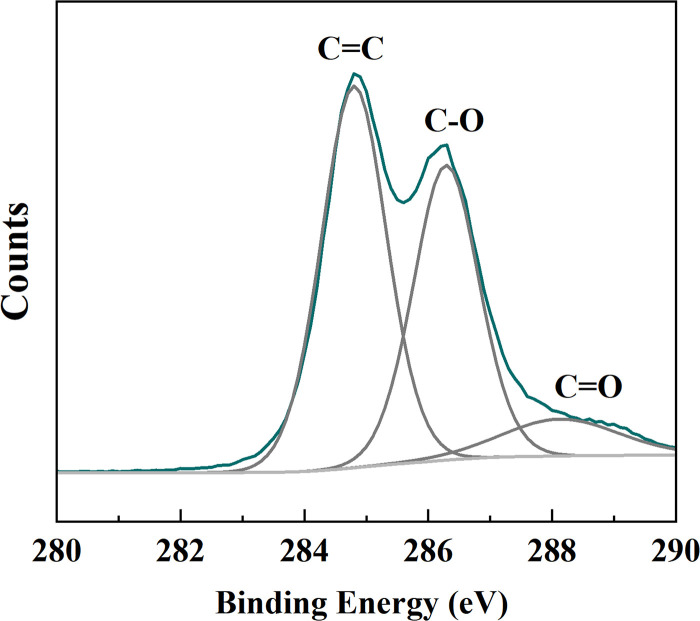
The C ^1^S XPS spectrum of the Gd@C_82_-TEGs nanoparticles.

The C ^1^S XPS spectrum of Gd@C_82_-TEGs nanoparticles exhibited three well-resolved peaks at binding energies of 284.8 eV, 286.3 eV, and 289.0 eV, which were assigned to C=C–C, C–O, and C=O species, respectively (Fig 4). These XPS data uniformly indicated that the conjugated framework of the pristine C_82_ fullerene was opened and covalently bonded to the triethylene glycol (TEG) moiety through a nucleophilic addition pathway, resulting in a C_82_–O–R linkage, despite the coexistence of a considerable amount of ketone (C=O) functionality. After calculated according to ICP-AES, the loading Gd amount in Gd@C_82_-TEGs nanoparticles was 8.0 wt%.

### *In vitro* MRI performance

**Fig 5:**
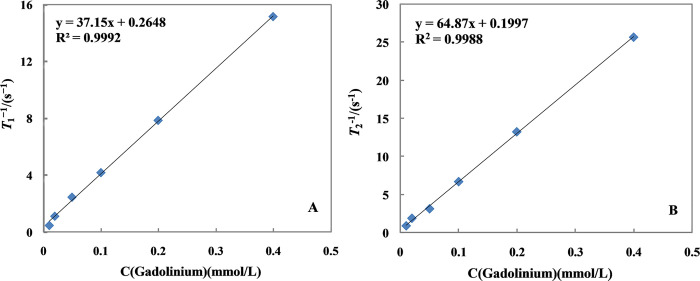
Plots of 1/*T*_1_ (A) or 1/*T*_2_ (B) *versus* Gd^3+^ concentration.

To verify whether Gd@C_82_-TEGs nanoparticles possess effective *T*_1_-weighted, the proton relaxivity (*r*_1_) and transverse relaxivity (*r*_2_) with different Gd^3+^ concentration were determined. The concrete data were seen in Fig 5.

It could be seen that the *r*_1_ value of the Gd@C_82_-TEGs nanoparticles was high up to 37.1 mM^−1^·s^−1^ in Fig 5A. The *r*_1_ value was far higher than that of the commercial Magnevist (Gd-DTPA, 3.19 mM^−1^·s^−1^). The appealing MRI enhancement ability of the Gd@C_82_-TEGs nanoparticles may attribute to the large area of the outer carbon cage, through which the embedded gadolinium atoms can efficiently and indirectly relax the water molecules. Moreover, the dipole-dipole interaction between the molecules further improved its relaxation efficiency. Notably, the transverse relaxivity (*r*_2_) was 64.9 mM^−1^·s^−1^ (Fig 5B), which greater than that of commercial Magnevist under the same conditions. Usually, the *r*_2_/*r*_1_ ratios of *T*_1_ agents were 1-2, while that of *T*_2_ agents is up to 10 or more. The *r*_2_/*r*_1_ ratio (*r*_2_/*r*_1_ = 1.74 < 2) at 0.50 T magnetic fields of Gd@C_82_-TEGs nanoparticles maybe beneficial for *T*_1_-weighted contrast effect.

**Fig 6:**
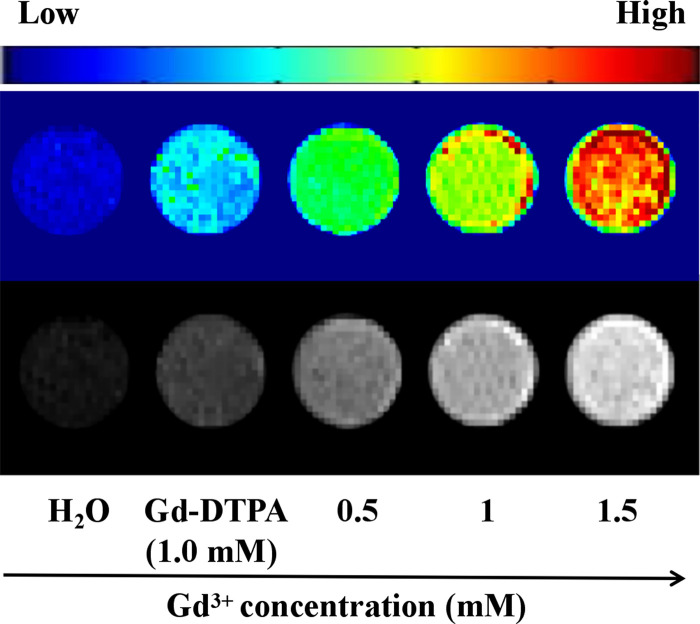
*T*_*1*_-weighted MRI of Gd@C_82_-TEGs nanoparticles at varied [Gd^3+^] (0 - 1.5 mM) and Gd-DTPA(1.0 mM) in H_2_O.

*T*_*1*_-weighted MRI of Gd@C_82_-TEGs nanoparticles at varying Gd^3+^ concentrations ranging from 0 to 1.5 mM and Gd-DTPA(1.0 mM) in H_2_O were shown in Fig 6. Under the same Gd^3+^ concentration, the MRI signal-enhancing efficiency of Gd@C_82_-TEGs nanoparticles was superior to that of Gd-DTPA. The results were in agreement with the proton relaxivity analysis.

### *In vitro* cytotoxicity of the Gd@C_82_-TEGs nanoparticles

**Fig 7:**
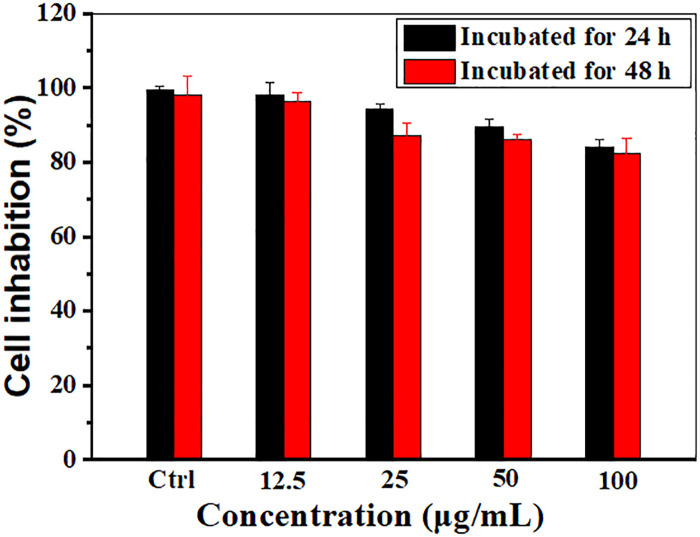
Cytotoxicity of the Gd@C_82_-TEGs nanoparticles in MCF-7 cells after 24 and 48 h incubation.

The *in vitro* cytotoxicity of the Gd@C_82_-TEGs nanoparticles against human breast cancer MCF-7 cells was assayed by MTT. It could be observed that there was no cell toxicity through 24 h and 48 h incubation in Fig 7. The relative viability remained above 80% in the concentration range of 12.5–100 mg/L. Even after incubation for 48 hours at a concentration of 100 mg/L, it remained as high as 82%.

**Fig 8:**
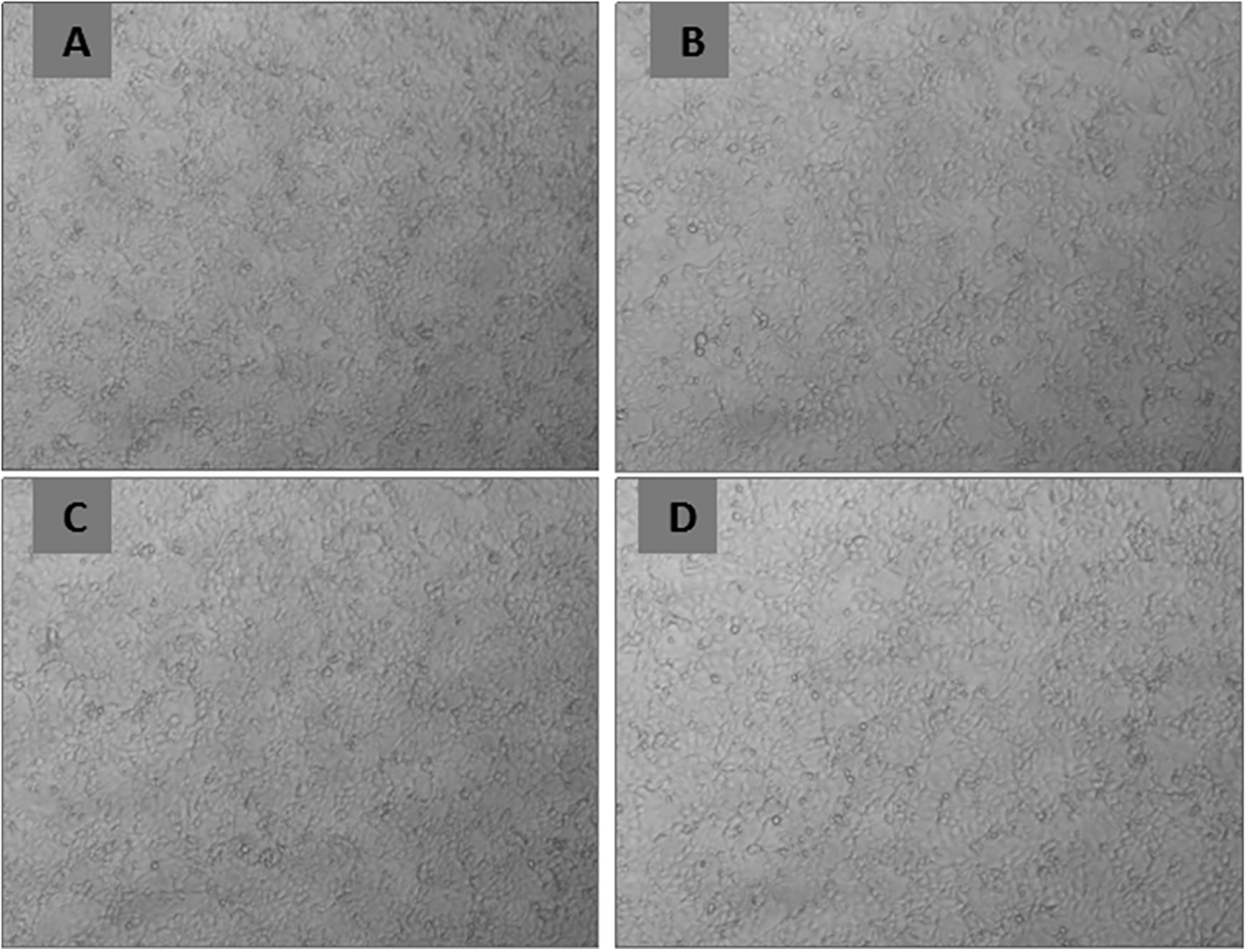
Morphology of MCF-7 cells with Gd@C_82_-TEGs nanoparticles (0- 100 μg/mL).

The morphology of MCF-7 cells was observed by a microscope. As shown in Fig 8, the morphology and structure of each group MCF-7 cells did not change after incubated with different concentrations (0, 25, 50, and 100 mg/L) of Gd@C_82_-TEGs nanoparticles for 48 h, and the morphology of MCF-7 cells in each group remained well. This indirectly indicated that the fullerene carbon cage could encapsulate the gadolinium atoms well, and the preparation method of Gd@C_82_-TEGs nanoparticles cannot change the structure of the fullerene so that the Gd@C_82_-TEGs nanoparticles will not produce obvious toxicity to cells.

## Conclusion

The water-soluble Gd@C_82_-TEGs nanoparticles as a novel MRI contrast agent were synthesized by a simple, effective and environmentally friendly self assembly method. The 97 nm spherical shape of Gd@C_82_-TEG nanoparticles ensured high relaxivity. The proton relaxivity (*r*_*1*_) and transverse relaxivity (*r*_*2*_) were high up to 37.1 mM^−1^·s^−1^ and 64.9 mM^−1^·s^−1^, which greater than that of commercial Magnevist under the same conditions. The obtained Gd@C_82_-TEGs nanoparticles showed noticeable *T*_*1*_-weighted MRI *in vitro*. Besides, the *in vitro* cytotoxicity experiments confirmed that the Gd@C_82_-TEGs nanoparticles had no significant effect on cell metabolism and structure, and the survival rate of cells was more than 82% after incubation for 48 hours at a concentration of 100 mg/L, which indicated that the as-prepared Gd@C_82_-TEGs nanoparticles were no cytotoxicity. The Gd@C_82_-TEGs nanoparticles are expected to serve as efficient MRI contrast agents for biomedical imaging.
